# Community Epidemiology Framework for Classifying Disease Threats

**DOI:** 10.3201/eid1112.050306

**Published:** 2005-12

**Authors:** Andy Fenton, Amy B. Pedersen

**Affiliations:** * Institute of Zoology, London, United Kingdom; † University of Virginia, Charlottesville, Virginia, USA

**Keywords:** Disease reservoirs, emerging communicable diseases, population dynamics, disease transmission, perspective

## Abstract

Recent evidence suggests that most parasites can infect multiple host species and that these are primarily responsible for emerging infectious disease outbreaks in humans and wildlife. However, the ecologic and evolutionary factors that constrain or facilitate such emergences are poorly understood. We propose a conceptual framework based on the pathogen's between- and within-species transmission rates to describe possible configurations of a multihost-pathogen community that may lead to disease emergence. We establish 3 dynamic thresholds separating 4 classes of disease outcomes, spillover, apparent multihost, true multihost, and potential emerging infectious disease; describe possible disease emergence scenarios; outline the population dynamics of each case; and clarify existing terminology. We highlight the utility of this framework with examples of disease threats in human and wildlife populations, showing how it allows us to understand which ecologic factors affect disease emergence and predict the impact of host shifts in a range of disease systems.

## Abstract 2

Ecologic and evolutionary features of multihost pathogens determine the likelihood of emerging infectious diseases.

Models of host-pathogen dynamics have typically assumed a single-host population infected by a single pathogen. However, most pathogens can infect several host species; >60% of human pathogens, >68% of wild primate parasites, and >90% of domesticated animal pathogens infect multiple host species (*1**–**3*). An interest in multihost pathogens is particularly timely, given that many of the most threatening current pathogens (e.g., HIV, West Nile virus, influenza virus, Ebola virus) are believed to have crossed species barriers to infect humans, domesticated animals, or wildlife populations (*1**,**3**–**8*). However, we do not know the host and pathogen characteristics that determine such host shifts and the likely characteristics of future emerging infectious diseases. To address this issue, 2 theoretical approaches have been adopted. The first, using dynamic models, focuses on the host's perspective and ascertains how a shared pathogen affects the dynamics of 2 host populations (*9**–**12*). The second approach takes the pathogen's point of view and considers how combined host densities affect pathogen persistence within the community (*13**–**15*). However, as the number of studies grows, so does the terminology. Terms such as multihost pathogens, dead-end hosts, reservoir hosts, host shifts, and spillovers are frequently used, but often different phrases are used to describe the same phenomenon, and possibly more concerning, the same terminology may be used to describe strictly different phenomena.

This lack of consolidation makes it unclear how these different approaches relate in terms of understanding the mechanisms driving disease emergence. A need exists for a single, comprehensive framework that characterizes disease outcomes based on biologically meaningful processes. Recently, attempts have been made to reconcile these concepts, mainly by highlighting the role of reservoir hosts (*13**,**16*). Haydon et al. (*13*) proposed a conceptual model that assumed a target host species was exposed to a pathogen endemic in a second host species (or species complex). The outcome of infection then depended on the sizes of the populations and whether they were able to maintain the pathogen alone. This approach expanded the naive view that reservoirs are nonpathogenic, single-species populations and encompassed the complexity of pathogen-host communities observed in nature. However, focusing just on host density ignores many key features of emerging diseases. The likelihood of disease emergence will depend on highly dynamic processes determined by both between- and within-species transmission rates. Therefore, ecologic forces acting on both hosts and pathogens will influence the contact structure of the community and affect the likelihood and persistence of an emerging infectious disease in a new host.

We propose a conceptual framework to describe the configurations of a host-pathogen community that may lead to disease emergence in a target host. We develop our framework from a simple 2-host 1-pathogen model and establish thresholds for pathogen and host persistence based on the between- and within-species net transmission rates. We then consider what ecologic factors determine the location of various host-pathogen systems within the framework. Finally, we use a stochastic model to consider what characteristics of the hosts and pathogen define the dynamics and likelihood of an emerging infectious disease.

## Conceptual Framework of an Emerging Infectious Disease

**Figure 1:**
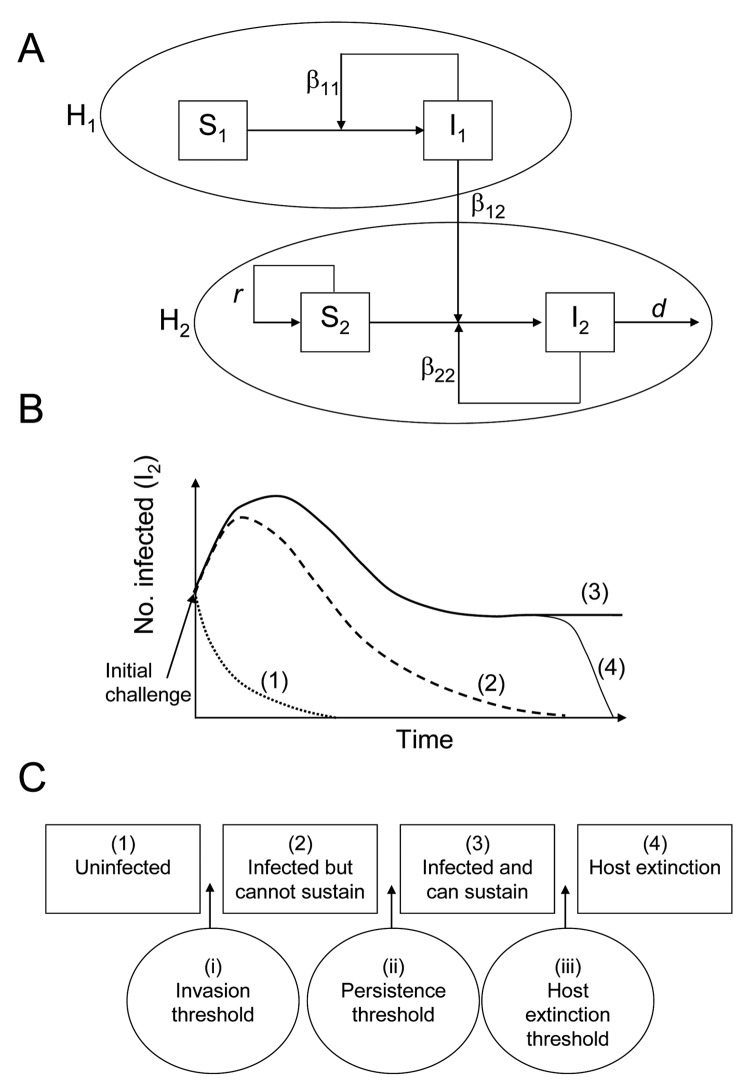
Emerging infectious disease framework. A) Schematic diagram of the multihost-pathogen community. B) Possible outcomes for a novel host, *H*_2_, after an initial infection by a pathogen endemic in an existing host, *H*_1_, where (1) the pathogen is unable to invade *H*_2_, (2) the pathogen invades but cannot be sustained within *H*_2_, (3) the pathogen invades and persists in *H*_2_, and (4) the pathogen invades and drives *H*_2_ to extinction. C) Three thresholds separating the 4 possible outcomes: (i) the invasion threshold, (ii) the persistence threshold, and (iii) the host extinction threshold.


We start by considering the assembly of a 2-host community infected by a single pathogen (*15**,**17**,**18*) where the pathogen is endemic within host population *H*_1_ such that individuals of *H*_1_ are either susceptible (*S*_1_) or infected (*I*_1_). We then assume a second target host population (*H*_2_) enters the community and can become infected by the pathogen (Figure 1A). Since the pathogen is well established in *H*_1_, we assume *S*_1_ and *I*_1_ are unchanged by *H*_2_; thus, our model most closely resembles the asymmetric model of Dobson (*15*). In the terminology of Haydon et al. (*12*), *H*_1_ is a maintenance host species (or species complex) with the potential to be a disease reservoir for *H*_2_. *H*_2_ may or may not be a maintenance host (see below). The model is

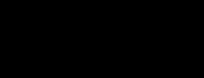
 (model 1)where *r* is the reproductive rate, *K* the carrying capacity, and *d* the death rate of the infected hosts. The composite functions *f*_22_ and *f*_12_ describe the net within-species (*H*_2_ to *H*_2_) and between-species (*H*_1_ to *H*_2_) transmission rates, respectively. We assume density-dependent transmission and so these functions have the form *f_ij_* = β*_ij_ I_i_ S*_2_, where β*_ij_* is the per capita transmission rate from species *i* to species *j*. Therefore, for example, the net rate of transmission from *H*_1_ to *H*_2_ (*f*_12_) depends on the size of the susceptible target population (*S*_2_), the size of the reservoir (*I*_1_), and the level of exposure and susceptibility of *H*_2_ (β_12_).

The target host population *H*_2_ has 4 possible outcomes: 1) uninfected, 2) infected but unable to sustain the pathogen, 3) infected and able to sustain the pathogen, or 4) infected and driven to extinction by the pathogen (Figure 1). These 4 outcomes are separated by 3 thresholds (Figure 1C): i) invasion threshold, ii) persistence threshold, and iii) host extinction threshold. The first 2 thresholds are analogous to established density-based thresholds in epidemiology; the first allows ecologic invasion of a pathogen, which subsequently dies out, and the second allows persistence of the pathogen (*19*). Here we combine these density effects with the per capita rates of infection to express these thresholds in terms of the magnitude of the net between- and within-species transmission rates (*f*_12_ and *f*_22_, respectively).

## Community-Epidemiology Continuum

**Figure 2:**
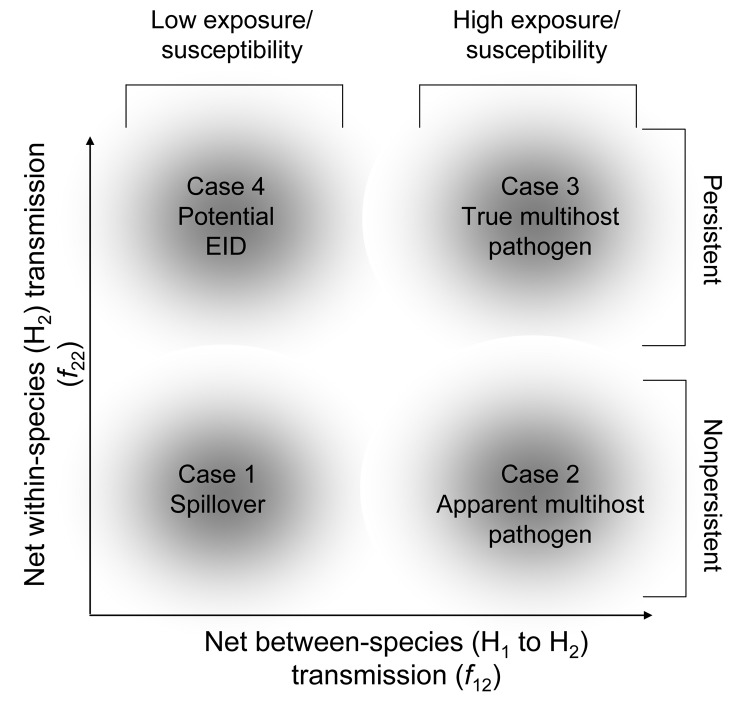
Community-epidemiology continuum, determined by the net between-*H*_1_ and -*H*_2_ transmission rate (*f*_12_) and the net within-*H*_2_ transmission rate (*f*_22_). EID, emerging infectious disease.

Infection of *H*_2_ by *H*_1_ and transmission within *H*_2_ are 2 separate processes determined by *f*_12_ and *f*_22_. Different combinations of these parameters lead to the different outcomes described above, and all possible scenarios can be placed within a 2-dimensional continuum (Figure 2), with *f*_12_ on one axis (i.e., can *H*_2_ get infected from *H*_1_?) and *f*_22_ on the other (i.e., can *H*_2_ sustain infection?). We can then divide the *f*_12_ – *f*_22_ parameter space into regions of different disease outcomes.

### Case 1: Spillover

In this case, the within-*H*_2_ transmission rate is too low to sustain the pathogen (*f*_22_ → 0). The between-species transmission from *H*_1_ is also low (*f*_12_ → 0). Thus, although infections of *H*_2_ do occasionally occur, they are transient. This represents the case in which the pathogen is specialized to the endemic host and there is either very low exposure to *H*_2_ (an ecologic constraint, such as parasite transmission mode) or *H*_2_ is resistant to infection (a physiologic constraint). We recommend the term spillover to describe this form of cross-species infection. Previously, spillover has been used to describe a wide range of dynamics (*20*), but we recommend limiting its use to transient infections in a target host because of transmission from a reservoir host that is not self-sustaining in the target population.

The recent outbreak of West Nile encephalitis in the United States is such a spillover: the virus moved from bird populations (*H*_1_) to infect humans (*H*_2_), which are unable to transmit the pathogen (β_22_= 0) (*21*). Nevertheless, spillovers still represent a serious health concern; increases in the reservoir population may lead to dramatic increases in disease prevalence in the target host.

### Case 2: Apparent Multihost Pathogen

In this case, the within-species transmission rate for the target host is low, but the between-species transmission rate exceeds the invasion threshold, resulting in persistent infections in *H*_2_. This case represents apparent multihost dynamics that differ from spillover dynamics in that the disease is nontransient in *H*_2_, but the pathogen is sustained because of frequent between-species transmission from the disease-endemic host. Apparent multihost dynamics exist because the potentially high prevalence in the target host would give the appearance of a true multihost pathogen, but the lack of within-species transmission means the disease cannot be maintained in the absence of *H*_1_. We recommend the term reservoir to describe *H*_1_ in both cases 1 and 2, in which the pathogen is permanently maintained in *H*_1_ and without between-species transmission (β_12_), the disease would not persist in the target host.

An example of an apparent multihost pathogen is rabies in side-striped jackals (*H*_2_) in Africa. Until a recent analysis (*22*), rabies was considered sustainable in the jackal population (*H*_2_), but detailed monitoring showed that rabies is not self-sustaining because of the density of the low susceptible jackal population (*S*_2_), and epidemics are frequently seeded from the domestic dog reservoir (high β_12_).

### Case 3: True Multihost Pathogen

In this case, both the within- and between-species transmission rates are high. Thus, since the pathogen can independently persist in either host population in the absence of the other, following Haydon et al (*13*), both are considered maintenance hosts. This case represents a true multihost pathogen with substantial within- and between-species transmission. One example is brucellosis infections around Yellowstone National Park, where the pathogen can be endemically maintained in cattle, bison, and elk populations (*23*).

### Case 4: Potential Emerging Infectious Disease

In this case, the within-*H*_2_ transmission rate is high, but the between-species transmission rate is very low (*f*_12_ → 0). Thus, the pathogen can persist in the target host (*H*_2_), but the net rate of between-species transmission is so low that *H*_2_ is rarely exposed to the disease. This case might occur when a disease is transmitted through close contact and thus has little chance of transmission between species. Similarly, the barrier to infection could be an ecologic factor, such as geographic isolation, which may be overcome by an anthropogenic change such as the introduction of exotic or invasive species. Thus, this case represents a potential emerging infectious disease in which the pathogen will become self-sustaining in *H*_2_ once the initial barrier to infection has been crossed. This case may be the region of greatest future concern since a single transmission event can have devastating consequences because of the high rate of within-species transmission in the target host.

Recent examples of potential emerging infectious diseases that were realized include the emergence of HIV-1 and HIV-2 in human populations, in which the close-contact nature of the infection process prevented transmission of simian immunodeficiency virus (SIV) from primates to humans (*6**,**24*). Another example is severe acute respiratory syndrome–associated coronavirus in humans, in which the primary transmission event is believed to be the result of close human contact with civet cats in China. Once the infection was successful, it spread rapidly throughout the human population by direct contact (*25*).

## Factors Affecting Location of a Host-Pathogen Community

The location of a host-pathogen system within the continuum will be determined by characteristics of both host populations and the pathogen. For instance, the pathogen's transmission mode will greatly determine its likelihood of encountering new hosts (*26*). Parasites transmitted by close contact may have limited exposure to multiple species and thus transmission modes that decouple host-to-host contact (i.e., waterborne or soilborne transmission) will increase the opportunity for between-species transmission. Evidence from wild primates and humans shows that pathogens with direct contact transmission are associated with high host specificity (*1**,**3*). Therefore, host-pathogen systems should segregate along the *f*_12_ axis according to their transmission mode.

Furthermore, the evolutionary potential of a pathogen will affect its ability to infect a new host (*2**,**27*). Pathogens in taxa with high mutation rates, antigenic diversity, and short generation times may rapidly adapt to new hosts (*28**,**29*), and recent evidence suggests that RNA viruses are the most likely group to emerge in humans (*26**,**30*), possibly because of their high mutation rate (*31*). Thus, host-parasite systems may segregate along the *f*_22_ axis according to taxonomy. Similarly, the phylogenetic relationship between the reservoir and target host will have consequences for disease emergence; viruses are less likely to jump to new hosts as the phylogenetic distance between hosts increases (*32*).

However, host-pathogen systems are not static, and a community may move across the continuum either because of ecologic or evolutionary shifts of the host or pathogen (*27*). In particular, anthropogenic changes, such as environmental exploitation and the introduction of domestic animals into previously uninhabited areas, may increase exposure to the pathogen and drive such transitions. For instance, although transmission of SIV from chimpanzees to humans may have occurred on a number of distinct occasions (*6*), these spillovers remained isolated. Only through various anthropogenic changes, including urbanization (increasing *S*_2_) and increased global travel (increasing β_22_) did the HIV pandemic take off in the 20th century.

In addition, pathogen evolution may greatly affect the likelihood of disease emergence by increasing the pathogen's basic reproductive ratio (*R*_0_) (*18**,**26*). For example, avian influenza has emerged several times in human populations since 1997. Typically, limited human-to-human transmission exists (β_22_ ≈ 0), so that although the avian reservoir (*I*_1_) and susceptible human populations (*S*_2_) are high, outbreaks are rare and isolated (i.e., occupying region 1 of the continuum). Only through recombination between strains and acquisition of human-specific respiratory epithelium receptors (thereby increasing β_22_) could the virus evolve sufficient transmissibility to be sustained in the human population, which poses the greatest risk for pandemics (*33*). These genetic changes could shift avian flu from being a spillover to becoming a true multihost parasite, which would have serious implications for human health.

## Stochastic Dynamics and Consequences for Vulnerable Host Populations

Theoretical and empiric evidence suggest that pathogens harbored by reservoir host populations are of particular concern because they can drive target hosts to extinction (*34*). Therefore, we must investigate population dynamic properties of different regions of the continuum and regions that pose the greatest risk for a target host. In a deterministic model, the invasion and persistence thresholds are the same and are determined by the pathogen's basic reproductive ratio (*R*_0_); if *R*_0_>1, an initial infection can both become established and persist. As shown by Dobson (15), *R*_0_ for a pathogen in an asymmetric host community (with no back-transmission from the target host to the reservoir) is dominated by the largest within-species transmission term, which implies that infection dynamics in the 2 host populations are largely independent; once between-species transmission has occurred, infection in *H*_2_ is driven solely by within-*H*_2_ transmission. However, in the stochastic reality of the natural world, an established infection may fade out, and reinfection from *H*_1_ could occur in the future (*19*). Therefore, we developed a stochastic analog of the above deterministic model to explore dynamics of the community-epidemiology continuum. The model was a discrete-time Monte Carlo simulation model, in which each event in model 1 (births, deaths, between- and within-species transmission) occurred probabilistically, and the next event was chosen at random based on those probabilities. The model was run 100 times for different combinations of within- and between-species transmission rates, and the infection status of the target host (*H*_2_) was measured as the mean prevalence over time, the proportion of time the pathogen was absent from *H*_2_ (the proportion of time the pathogen faded out), and the proportion of runs in which the pathogen drove the host to extinction. This stochastic model is appropriate for exploring the dynamics of emerging infectious diseases not captured by continuous-time deterministic models, in particular when exposure of a target host to a pathogen from a reservoir is likely to occur at discrete intervals (*27*).

**Figure 3:**
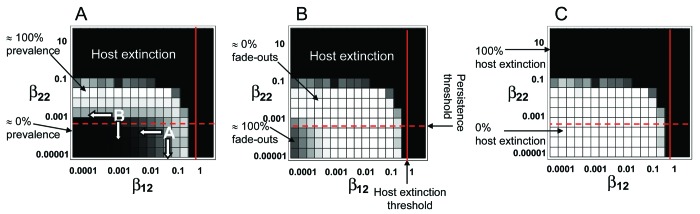
Stochastic model predictions of system behavior in β_12_–β_22_ parameter space. Each square represents the average of 100 simulation runs. Two measures of pathogen persistence are shown: A) Mean prevalence of infection in *H*_2_, where black represents zero prevalence and white represents 100% prevalence, and B) Proportion of time in which the pathogen is absent (i.e., has faded out) from *H*_2_, where white represents zero fade-outs (i.e., the pathogen is always present in *H*_2_) and black represents 100% fade-outs (i.e., the pathogen never infects *H*_2_). C) Probability of pathogen-driven host extinction, where black represents the case in which all runs resulted in host extinction and white the case in which none of the runs resulted in host extinction. The horizontal dashed lines are the deterministic approximations of the persistence threshold (given by *R*_0_ = 1 when β_12_ = 0) and the vertical lines are the deterministic approximations of the host extinction threshold. The points marked A and B in panel A and the associated arrows represent different control scenarios for 2-host pathogen systems located at different points within the continuum (see text for details).

As in the deterministic case, low between- and within-species transmission prevents the pathogen from persisting in the target host (prevalence ≈0, Figure 3A; proportion of time pathogen was absent ≈100%, Figure 3B). Increasing the exposure of *H*_2_ to the pathogen (i.e., increasing β_12_) leads to a gradual increase in both the prevalence of infection and the proportion of time the pathogen is present in *H*_2_. This increase applies even if within-*H*_2_ transmission is negligible (β_22_ → 0). Therefore, regular, high exposure to the pathogen from the reservoir can give the appearance of endemic infection, even if the pathogen cannot be sustained within the population (case 2: apparent multihost dynamics). Increasing the within-*H*_2_ transmission rate (β_22_) from very low levels has little impact on the prevalence of infection or the proportion of time *H*_2_ is infected. Eventually, however, a point is reached at which increasing β_22_ suddenly allows the long-term persistence of the pathogen in *H*_2_. At this point, the persistence threshold is reached and the pathogen becomes endemic in *H*_2_, regardless of input from *H*_1_. This threshold can be approximated from the deterministic model by setting β_12_ = 0 and solving for *R*_0_ = 1, which shows that β_22_ must be > (*d* + *r*)/*K* for the pathogen to persist in the absence of input from *H*_1_ (the horizontal line in Figure 3).

Increasing either between- or within-species transmission rates (β_12_ or β_22_) leads to a point when the host is driven to extinction (Figure 3C), which highlights the danger of an emerging infectious disease; even if *H*_2_ is a poor transmitter of the disease (β_22_ → 0), repeated exposure from H_1_ may be sufficient to drive the population to extinction. Analysis of the equivalent deterministic model (model 1) suggests that this threshold should be in the between-species transmission rate (β_12_) only (host extinction is not affected by β_22_) and is given by β_12_ > *dr*/(*d – r*) for *H*_2_ extinction to occur (shown by the vertical line in Figure 3). Thus, even if the probability that *H*_2_ will contract the pathogen is very low (β_12_ → 0), a single transmission event may spark an epidemic that completely decimates the population (region 3).

## Implications for Disease Control

The correct classification of the different regions of the community-epidemiology continuum are of more than just semantic importance; quantifying the between- and within-species transmission rates and the location of a host-pathogen system within the continuum are vital to determine the appropriate control strategy. Haydon et al. (*13*) proposed 3 means of controlling infection in a target-reservoir system: 1) target control, which is aimed at controlling infection within the target population; 2) blocking tactics, to prevent transmission between the reservoir and target host population; and 3) reservoir control, which suppresses infection within the reservoir. These 3 control strategies correspond to reducing the within- and between-species transmission rates (β_22_, β_12_, and β_11_, respectively). The benefits of each approach will vary according to the relative contributions different transmission processes make to the overall prevalence in the new host (*H*_2_). Our stochastic model showed that high exposure to the pathogen from the reservoir host can give the appearance of endemic infection in the target host, even if it cannot sustain the pathogen alone. In this case, the optimal control strategy is completely different from that used against a true multihost pathogen endemic in the target host. For a host-pathogen system in region 2 of the continuum (apparent multihost dynamics), where between-species transmission rates are high but within-*H*_2_ transmission rates are low (point A in Figure 3A), the prevalence of infection in *H*_2_ may be very high, but mounting a target control program aimed at reducing within-*H*_2_ transmission is unlikely to be effective (the vertical arrow from point A in Figure 3A). However, blocking control, which would reduce transmission from the reservoir to the target host, may drastically reduce prevalence (the horizontal arrow from point A in Figure 3A). Conversely, similar levels of prevalence in *H*_2_ may be observed for a host-pathogen system located in region 4 of the continuum (point B in Figure 3A) but because of fundamentally different processes. In this case, blocking tactics aimed at preventing transmission from the reservoir to the target host will be ineffectual (horizontal arrow from point B in Figure 3A), but target control may prove highly effective (vertical arrow from point B in Figure 3A). Therefore, establishing the initial location of a novel host-pathogen system within the community-epidemiology continuum and understanding the within- and between-species transmission rates are essential for optimizing vaccination and culling strategies to lessen the impact of disease.

## Conclusions

This report provides a conceptual framework to understand the ecologic characteristics of disease emergence based on between- and within-species transmission rates involving a potential disease reservoir population and a target host population. Using this framework, we outlined 4 possible cases of long-term disease dynamics in the target host and showed that these outcomes occupy different regions of a 2-dimensional continuum described by the net between- and within-species transmission rates. Furthermore, the development of the community-epidemiology framework allows us to clarify the wealth of terminology currently used to describe disease occurrence in host communities, based on an understanding of the underlying ecologic and epidemiologic processes. In particular, the much-overused terms reservoir and spillover can be seen to have explicit definitions, depending on whether the pathogen can be sustained within the target host population.

By explicitly considering how the ecologic and evolutionary characteristics of hosts and pathogens combine to affect the between- and within-species transmission rates, and the subsequent consequences for disease occurrence in a novel host, this framework highlights that current human diseases, domestic and wild animal diseases, and the threats of emerging infectious diseases can be understood by a quantitative framework of the underlying transmission processes. Given that most parasites can infect multiple host species and the recent surge of emerging infectious diseases in wildlife and human populations, understanding the dynamics of disease persistence in novel hosts has never been more important.
